# Impact of socioeconomic position on childhood obesity in Finland based on register data from 2018

**DOI:** 10.1093/eurpub/ckac130.044

**Published:** 2022-10-25

**Authors:** L Paalanen, E Levälahti, P Mäki, H Tolonen, T Laatikainen

**Affiliations:** 1 Department of Public Health and Welfare, Finnish Institute for Health and Welfare, Helsinki, Finland; 2 Institute of Public Health and Clinical Nutrition, University of Eastern Finland, Kuopio, Finland

## Abstract

**Background:**

Obesity is a globally growing public health challenge among children. In developed countries, the risk of obesity is commonly higher among lower socioeconomic groups. Measuring socioeconomic position (SEP), especially income, is challenging in surveys as self-reported information may suffer from reporting, awareness, recall and non-response bias. Our aim is to utilize official register data on several SEP indicators and measured height and weight of children, to identify the strongest predictors of SEP of the parents on the risk of obesity among the whole 2-17-year-old child population in Finland.

**Methods:**

Data for all children who had visited child health clinic or school health care in 2018 were extracted from the National Outpatient Register on Primary Health Care Services (n = 387623, coverage 40% in 2018). Obesity was defined according to the WHO criteria. SEP indicators were obtained from Statistics Finland for both parents living in the same household with a child. Boosted regression model was used to analyze the contribution of SEP to obesity using training dataset on 155479 non-related children.

**Results:**

The area under the curve for the final model in training dataset was 0.736 and 0.718 in validation dataset. Mother's educational level (12.6% of the total explained variation) and household's disposable income (12.6%) were the SEP indicators that most strongly predicted childhood obesity, whereas the impact of educational level of the father was somewhat smaller (8.1%). The influence of the age of a child was even bigger (39.2%), the prevalence of obesity being highest at 11 and 9 years of age among boys and girls, respectively.

**Conclusions:**

Our results based on official register data from Finland, a Nordic high-income country, endorse earlier findings on higher obesity risk among children with low socio-economic family background. Identification of the SEP related risk factors and support to families are essential in the prevention of childhood obesity.

**Key messages:**

- Several family socio-economic factors are reflected in the risk of childhood obesity.
- Utilizing objective register data offers a way to tackle many challenges related to self-reported survey data.

